# Persistence of the otoprotective effect. How long does otoprotection against amikacin lasts?

**DOI:** 10.5935/1808-8694.20120032

**Published:** 2015-10-20

**Authors:** Andreia Ardevino de Oliveira, Matheus de Souza Campos, Adriana de Andrade Batista Murashima, Maria Rossato, Miguel Angelo Hyppolito, José Antônio Apparecido de Oliveira

**Affiliations:** a PhD in Otorhinolaryngology - School of Medicine of Ribeirão Preto - University of São Paulo (USP) - Department of Ophthalmology, Otorhinolaryngology and Head and Neck Surgery. Assistant Physician - University Hospital - Medical School of Ribeirão Preto - USP); b MD. ENT - School of Medicine of Ribeirão Preto - University of São Paulo (USP) - Department of Ophthalmology, Otorhinolaryngology and Head and Neck Surgery; c MSc student in Medical Sciences - School of Medicine of Ribeirão Preto - University of São Paulo (USP) - Department of Ophthalmology, Otorhinolaryngology and Head and Neck Surgery - Lab Technician; d Lab Technician; e PhD in Medical Sciences; Professor at the School of Medicine of Ribeirão Preto - University of São Paulo (USP) - Department of Ophthalmology, Otorhinolaryngology and Head and Neck Surgery; f Full Professor - retired - School of Medicine of Ribeirão Preto - University of São Paulo (USP) - Department of Ophthalmology, Otorhinolaryngology and Head and Neck Surgery

**Keywords:** amikacin, aminoglycosides, drug toxicity, hair cells, auditory, inner, hair cells, auditory, outer

## Abstract

There is evidence that a “resistance phenomenon” occurs when a none-damaging dose of amikacin protects the hair cells from ototoxicity. Our goal is to prove that this resistance is persistent.

**Method:**

Experimental study - 14 albino guinea pigs (*Cavia porcellus*) divided into three groups. The auditory function was assessed by distortion product otoacoustic emissions (DPOAE): before exposure to amikacin, on the 15th day after the non-damaging dose was injected, at the end of the damage dose injection and prior to decapitation.

**Results:**

Group A (control) presented normal hearing and histological pattern. Group B (amikacin 20 mg/kg/day (IM) for 30 days and affecting dose (400 mg/kg/day) for 12 days and Group C (same protocol of Group B, but kept for 60 days and slaughtered), the DPOAE confirmed normal auditory function in the pre-exposure and maintenance of the standard-dose; however, significant loss of auditory function after the end of the damaging dose injection.

**Conclusion:**

The protection phenomenon did not extended for a period of 30 to 60 days after the application of damaging doses of amykacin.

## INTRODUCTION

Aminoglycosides are among the most used antibiotic agents in the world because of their high efficacy and low cost. Nonetheless, they do have important side effects, such as kidney toxicity and ototoxicity.

The ototoxic action of these antibiotics happen directly on the polyphosphoinositides receptors, located on the membrane of hair cells of the organ of Corti, of the saccular and utricular macula and the ampullary crests of the vestibular system. These receptors are lipidic components of the cell membrane which form complexes with aminoglycosides, bringing about changes to the membrane permeability, which may cause cellular failure with consequent hearing loss^1^.

The ototoxicity mechanism against the hair cells involves the capture of these aminoglycosides by these cells, through receptor-mediated endocytosis^2^, individual genetic predisposition to cell damage involving mitochondrial DNA alterations^3^, ^4^, the damage action of Reactive Oxygen Species (ROS) - formed as a consequence of aminoglycosides scavenging iron^5^, ^6^ - all the way to programmed cell death by apoptosis^7^.

Ototoxicity may appear during acute or late exposure to aminoglycosides, even months after the exposure. It may evolve to a more severe degree or even recovery of normal auditory thresholds (prior to the exposure). When cochlear lesion ensues with destruction of the organ of Corti hair cells, hearing loss is irreversible^8^.

Amikacin, the first described aminoglycoside, is a derivative of kanamycin, active against most microbial species resistant to gentamicin and to kanamycin itself^9^.

The pattern of injury to the Organ of Corti involves the initial damage to the external hair cells on the basal turns of the cochlea, later progressing with a lesser degree of cochlear lesion, towards the cochlear apex^10^. The lesions affect preferably the outer hair cells, initially reaching the first row of cells, then following to the second and third rows^11^.

A dose of 400 mg/kg/day of intramuscular amikacin for 12 days causes the complete destruction of outer hair cells and a partial lesion to the internal ones, on the first and second turns of the cochlea of guinea pigs, with lesser lesions on the third and fourth turns^10^.

Non-damaging sound stimuli (low intensity ones), employed during a long period of time prior to the exposure to traumatic noise of the same type, protect the cochleas of lab animals, reducing the physiological alterations and the lesions to the sensorial cochlear cells^12^, a phenomenon known as resistance. It is very likely that the conditioning stimuli would change the cell, making it more capable of withstanding damaging stimuli and the protection seems to be mediated by cochlear changes^13^.

It has been proved that the resistance phenomenon is also manifested after the prior and longstanding administration of non-damaging doses of amikacin before employing the ototoxic doses, in other words, the non-damaging dose of amikacin significantly protects the hair cells against the ototoxicity of amikacin itself on the two most basal turns^14^.

Our goal has been to study the otoprotection of the outer hair cells against the ototoxicity caused by amikacin is temporarily persistent.

## METHOD

We utilized fourteen albino lab animals (*Cavia porcellus*), weighing 250g and with the Preyer's reflex. The study was approved by the Ethics Committee in experimentation with animals of our institution, approval protocols # 075/2008.

The drug utilized in this study was intramuscular amikacin. The Preyer's reflex was tested daily, the same happened with the assessment of body weight until the maximum time before animal slaughtering.

The animals were distributed in three groups:

**Group A (control)** - Four guinea pigs injected with intramuscular distilled water for 30 days (two animals) and 60 days (two animals).
**Group B** - Five animals injected with intramuscular amikacin 20 mg/kg/day, for 30 days (protective dose) and, afterwards, a dose of 400 mg/kg/day of amikacin (damaging dose) for 12 days, the mean necessary time for the elimination of the Preyer's reflex. After that, the animals were kept for 30 days on a regular diet, and they were afterwards slaughtered.
**Group C** - Five animals in the same amikacin administration regimen from Group B were maintained for 60 days and then slaughtered.

The technique used to study the histopathology changes in this study was the scanning electron microscopy (SEM), with attention to the structural damages caused to the Organ of Corti on the different cochlear turns, especially to the outer hair cells which received different doses of amikacin.

The guinea pigs were anesthetized by inhaling ether and then they were beheaded, their temporal bones containing their bullae were removed. The apex and the round window were opened and, for fixation, we used glutaraldehyde at 2.5% in a 0.1% phosphate buffer (So-rensen) at 4°C. The microdissection carried out preserved the spiral lamina with the organ of Corti. This material was preserved for 12 hours in a 0.1M buffer solution, and it was reaffixed in an osmium tetroxide solution with 0.1M phosphate buffer for 1 hour at 4°C. The following stages were: dehydration in ethanol with drying using the critical point of liquid carbon dioxide in a BALTEC - CPD 030 -“CRITICAL POINT DRYER” device. The cochlear was then settled in a cylindrical specimen holder, fixed with carbon conductive paste and plated with gold (thin layer) through a vaporizer (BALTEC - SDC 050) for perfect visualization upon scanning electron microscopy.

The auditory function of the animals was assessed by means of Distortion Product Otoacoustic Emissions (DPOAE) using the ILO 92 CAE System from Otodynamics LLC, with 70 dB SPL intensity of the triggering stimulus for F1 and F2, at a ratio of F1:F2 = 1.217. The DPOAE were studied out in the following situations: pre-exposure to amikacin, on the 15^th^ day of administration of a non-damaging dose, at the end of the administration of the damaging dose and before slaughtering.

The DPOAE provide a functional comparison of the outer hair cells before and after the administration of the habituation dose and the damaging dose, providing information on the drug action on these cells during the entire period of the study, reducing the interference of the constitutional difference on the auditory function of each animal, enabling the true assessment of the action of amikacin on the outer hair cells.

## RESULTS

**Figure 1:**
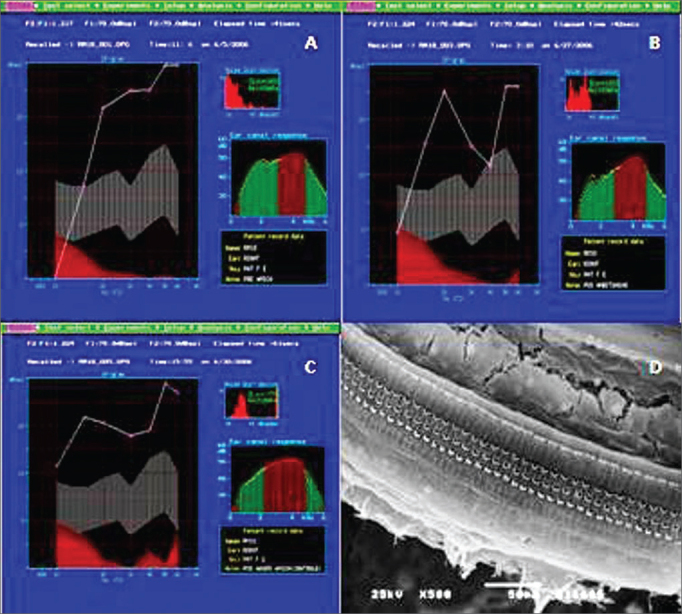
Example of DPOAE in group A. A: pre-exposure, we notice that the the auditory function is maintained, starting at 1 KHz, with the presence of DPOAE; B: After the non-damaging dose, showing the normal pattern with the presence of DPOAE; C: After the damaging dose, showing the presence of DPOAE; D: Example of SEM in the control group, showing normal inner and outer hair cells on the basal turn. 500x Magnifcation.

All the animals in Group A had normal auditory function and histological pattern. (Figure 1A-D).

**Figure 2:**
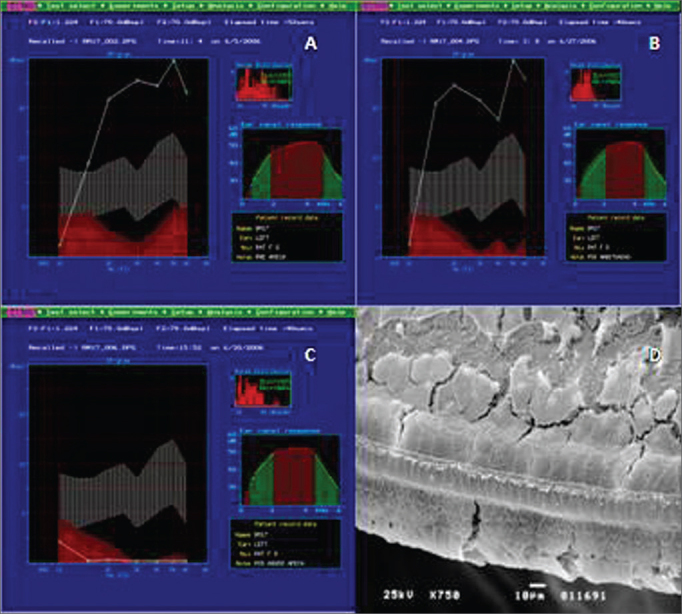
Example of DPOAE in Group B. A: pre-exposure, we notice that the auditory function was maintained, starting at 1 KHz, with the presence of DPOAE. B: DPOAE present after treatment with low, not damaging doses of amikacin. C: DPOAE present in all the tested frequencies after a damaging dose of 400 mg/kg/day of amikacin for 12 days. D: Example of SEM in Group B, showing the destruction of outer hair cells on the basal turn. 750x magnifcation.

In Group B, the study of DPOAE showed a normal auditory function before exposure to the drug and maintenance of the pattern after the non-damaging dose (Figure 2A-B). Nonetheless, we see an important loss of the auditory function after the end of the administration of the damaging nose, proven by the lack of DPOAE (Figure 2C).

In the histology exam (Figure 2D), we noticed the cochlear involvement. The Organ of Corti clearly lost its support, showing a damage pattern which started on the basal turn - usually the most affected region, with total absence of outer hair cells and the presence only of the inner hair cells - a pattern that is maintained in the E2 turn. On the E3 turn, the inner hair cells and the first and second rows of outer hair cells were the most affected, in such a way that we notice only the presence of cell extrusions in the row of inner hair cells and just a few intact cells on the third row of outer hair cells. On the apex turn, there are more intact cells on the 1^st^ row of outer hair cells, besides a lack of cilia on the inner hair cells.

In Group C, the functional findings assessed by the DPOAE and the histology exam by means of the SEM showed results identical to those found on Group B.

## DISCUSSION

The ototoxicity caused by aminoglycosides is one of the most common causes of preventable sensorineural hearing loss^15^.

The aminoglycosides have the capacity to interact with iron, generating an active metabolite that is capable of catalyzing the formation of ROS, which are highly reactive compounds, physiologically formed as a consequence of aerobic cell metabolism^16^. Among the main examples of ROS, we have the hydrogen peroxide and the hydroxyl, hydroperoxyl and superoxyde.

Such compounds may cause changes to the DNA, protein inactivation and lipid peroxidation of cell membranes. Despite such deleterious effects, these compounds are important for the body, because they also participate in the defense against pathogens^17^, ^18^. Therefore, it is indispensable to have a cell defense system against the potentially noxious actions of ROS, made up of antioxidant agents such as glutathione, catalase and superoxyde dismutase.

The co-administration of antioxidant agents to guinea pigs together with gentamicin yielded a reduction on the drug-induced hearing loss^19^.

## CONCLUSION

The self-defense phenomenon was not maintained beyond a period of 30 to 60 days after the injection of damaging doses of amikacin (400 mg/kg/day).

Cochlear otoprotection against the ototoxicity of aminoglycosides is proven; however, its mechanism is still not completely clear. We know that it probably involves the action of anti-free radical agents, such as glutathione, which is capable of inactivating the active metabolite formed by the interaction of amikacin and iron, which was found in the inner ear. Thus, the intracochlear levels of glutathione would be increased when low doses are administered for long periods of time before the administration of a damaging dose, protecting the cochlear OHC^14^.

The goal of the present study was to assess whether the mechanisms responsible for otoprotection would be able to maintain cell defense for longer periods of time -30 and 60 days. We can not state whether the self-defense phenomenon could last for more than 60 days.

We concluded that the high levels of anti free radicals which should protect the cochlea, were consumed during the period of time in which the animals were kept, thus generating a balance between the ROS (noxious agents) and the antioxidants (protective agents), allowing the noxious actions of the formers on the Organ of Corti. This unbalance is added to the high serum levels of amikacin (nephrotoxic effect).

This result helps outline new ways to better understand the phenomenon of self-defense, as intents to change the drug regimen, change the periods of administration of damaging doses or the periods of administration of the protective and damaging doses.

The attempt to better understand the cochlear self-defense mechanisms and to better act on maintaining such mechanisms for longer periods of time, until the end of the toxic effects of circulating aminoglycosides aims at developing a clinical applicability in pharmacological therapies.
